# Infected Nonunion of the Distal Femur in the Elderly with Bone Loss: Case Report and Treatment Options

**DOI:** 10.1155/2021/3530297

**Published:** 2021-09-17

**Authors:** Neven Starčević, Andrija Karačić

**Affiliations:** ^1^Traumatology Department, University Hospital Sveti Duh, Sveti Duh, 64 Zagreb, Croatia; ^2^Department of General Surgery, University Hospital Sveti Duh, Sveti Duh, 64 Zagreb, Croatia

## Abstract

The management of infected nonunion associated with bone loss in long bones is both a time-consuming and challenging procedure for the orthopedic and trauma surgeon. In this paper, we present the case of a 75-year-old woman with infected nonunion of the distal femur associated with bone loss after plate osteosynthesis for a distal femur fracture. The patient was referred for nonunion of the distal femur after plate fixation (nonlocking “classic” plate) and was treated with a locking compression plate (LCP) and autologous cancellous bone transplant. During the follow-up, the patient was ambulatory without pain; however, the nonunion failed to heal, therefore, the induced membrane technique (Masquelet procedure) was performed in two stages, tissue samples were taken and revealed a bacterial infection (*S. epidermidis*), and antibiotic treatment was started. Due to infection, fracture healing was slowed, but did commence. Unfortunately, the LC plate failed before union occurred, the nonunion was treated with a femoral nail and blocking (Poller) screws, and the bony defect was filled with Ca-P cement. The patient was operated one last time for cement dislocation when not only the dislocated cement was removed but also the femoral nail dynamized. After one year after treatment completion, the fracture healed, and leg length discrepancy was 1.5 cm shorter on the left side. The patient experienced significant pain relief and can walk with the help of crutches. Our paper demonstrates the application of different techniques in fracture surgery as they are required can result in fracture healing even in very adverse circumstances.

## 1. Background

Distal femoral fractures account for less than 1% of all adult fractures and between 3% and 6% of all femur fractures [1]. Their frequency among age groups is bimodally distributed: on one side, young patients after high-energy trauma, and on the other older, osteoporotic patients after low-energy trauma [2].

Due to their high severity, even today, distal femur fractures pose a great challenge even to the experienced trauma and orthopedic surgeons [3]. They are difficult to manage, and their treatment often leads to unsatisfactory results with nonunion rates as high as 34% [4].

Although there are several treatment options, there are no standard guidelines for the treatment of distal femur fractures. Possible treatment options are open reduction and internal fixation (ORIF) using locking compression plates (LCP), dynamic condylar screws, angled blade plates, and closed reduction and fixation with retrograde intramedullary nails (RIMN). The great diversity of treatment options merely reflects the great challenges associated with this type of fracture [1]. Surgeons often decide on the modality of treatment depending on their experience and knowledge and the tools and implants they have available.

The most feared complication in fracture surgery is nonunion which is associated not only with bone loss and deformity but also frequently with infection [5]. Risk factors for nonunion are not only patient dependent such as gender, age, and comorbidities but also features of the fractures itself: open fractures, intraarticular, or comminuted fractures [3].

The management of an infected nonunion in the distal femur remains one of the greatest challenges in fracture surgery [6]. Not only for the surgeon: distal femur fracture nonunion has a devastating impact on the patient's function and quality of life. We present a case report of a patient whose infected nonunion of distal femur fractures had been treated with different novel techniques such as the Masquelet procedure and revision of a failed locked plate with a locked femoral nail complicated by Ca-P cement dislocation. After a thorough literature review, we could not find any similar cases which mark our case as a technical novelty.

The following case report has been reported in line with the SCARE criteria [7].

## 2. Case Presentation

The study was conducted in accordance with the principles of the Declaration of Helsinki. The patient has signed informed consent about the treatment she was subjected to and the processing of her personal information.

**Figure 1:**
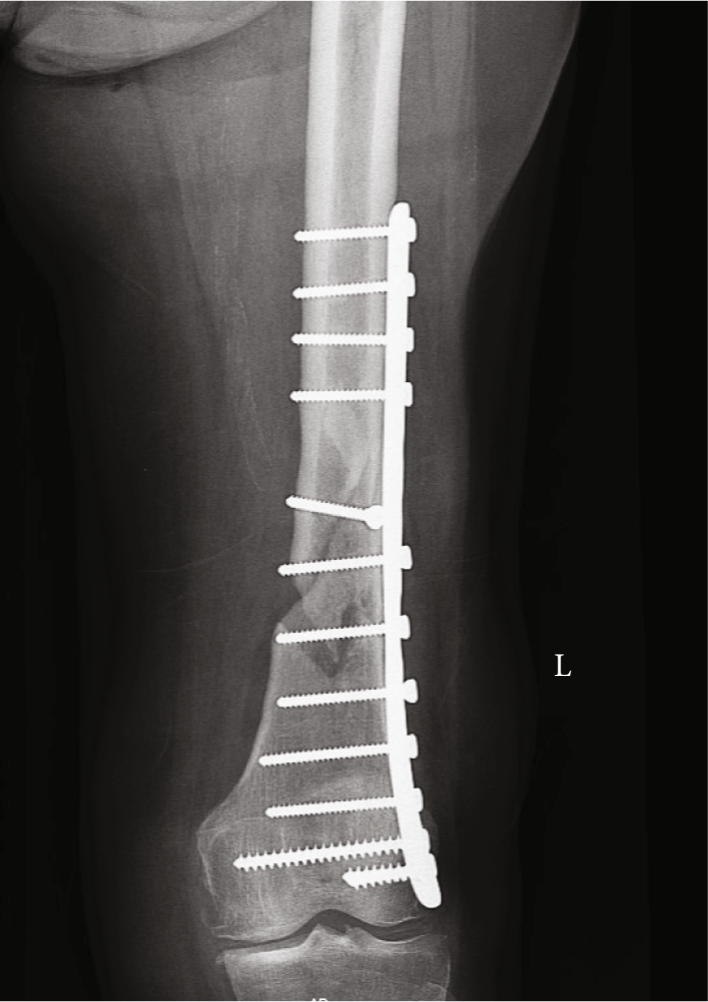
The visible nonunion with the classic plate in AP view.

**Figure 2:**
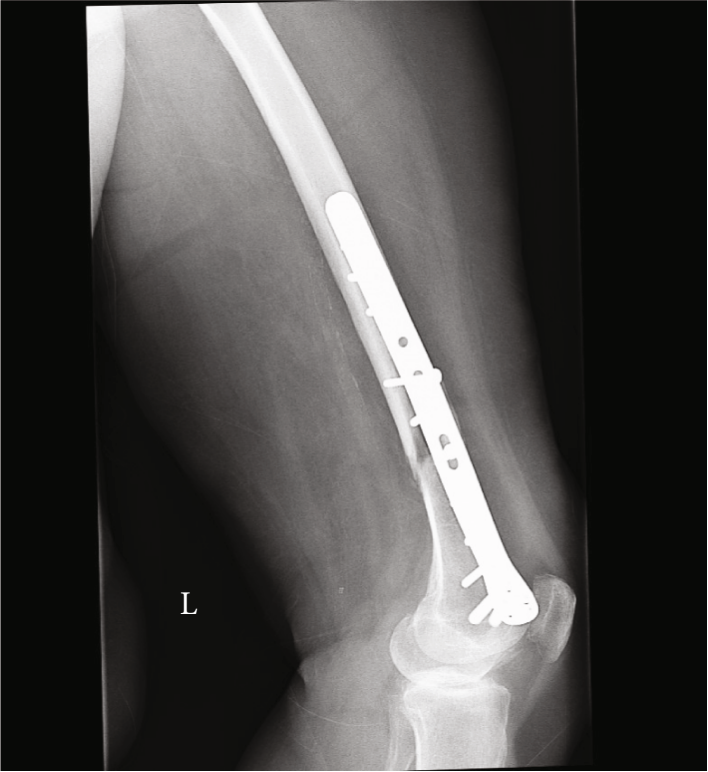
The visible nonunion with the classic plate in LL view.

We present the case of a 75-year-old woman who presented to our outpatient office in a tertiary care facility looking for a second opinion. The patient had been operated on 6 months before, presenting with a left distal femur fracture. Her comorbidities that included diabetes type 2 (well-regulated, on oral medication), arterial hypertension, sideropenic anemia, and medical history show cataracts and varicose vein surgery. Her medical history did not include any diagnostic work-up for osteoporosis. Clinically, the distal left thigh appeared normal on inspection, and palpation revealed tenderness of the delayed union. The range of motion was normal. X-ray showed an atrophic nonunion after “classic” plate fixation of the distal femur fracture with bone loss (Figures 1 and 2).

**Figure 3:**
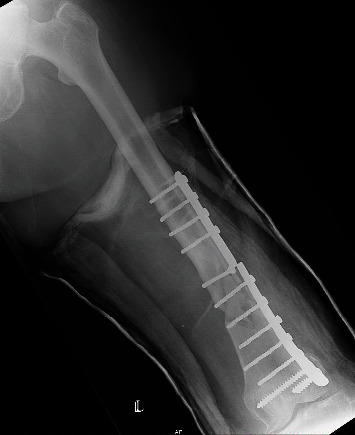
The nonunion with the failed classic plate in AP view.

A reosteosynthesis with autologous cancellous bone transplant was recommended. Unfortunately, a month after the visit, the patient was presented to the emergency room with acute onset pain and instability in the left lower extremity after walking. Classic X-ray showed plate failure and fragment dislocation (Figure 3).

**Figure 4:**
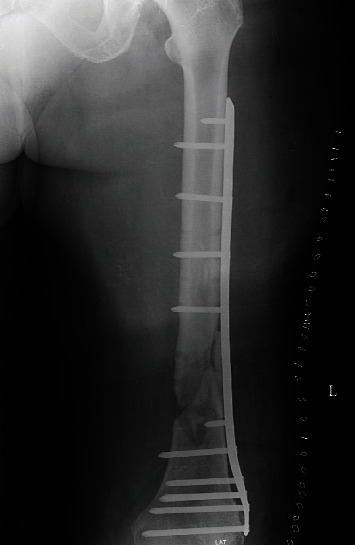
The debrided nonunion with autologous cancellous bone graft stabilized with an LCP in AP view.

**Figure 5:**
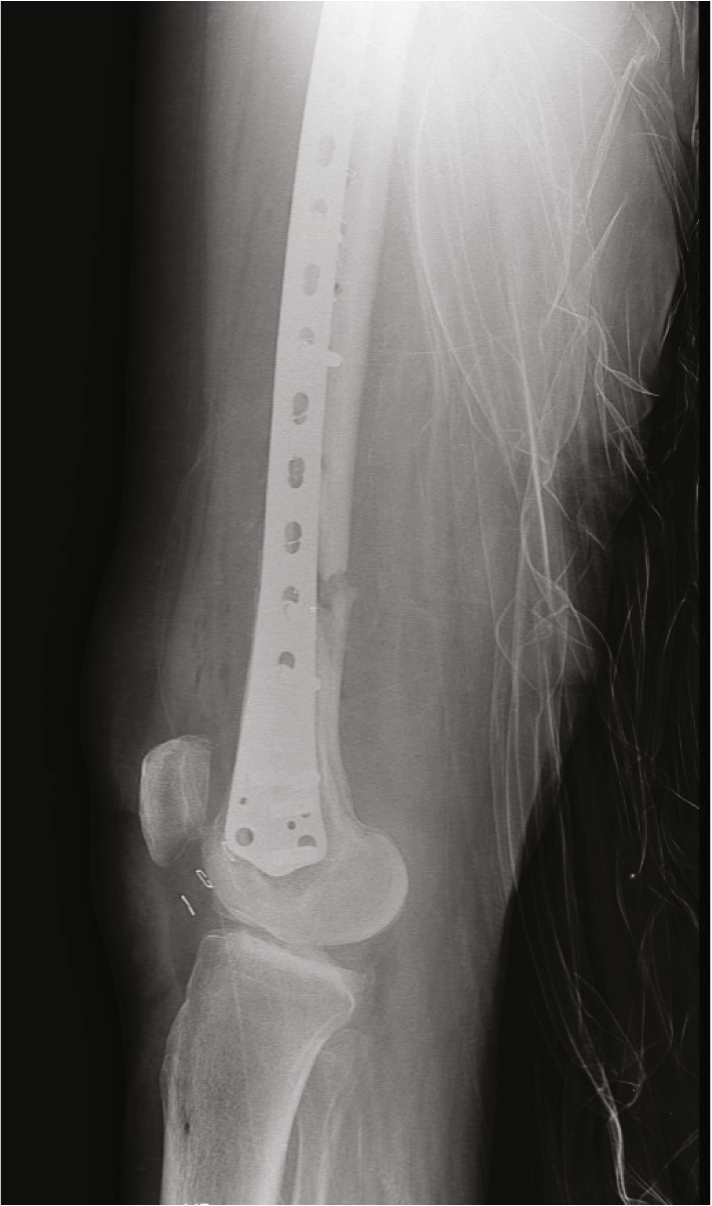
The debrided nonunion with autologous cancellous bone graft stabilized with an LCP in LL view.

The patient was admitted to the hospital, and surgery was performed after written/informed consent: the first plate was removed, a reosteosynthesis with a titanium locking compression plate was performed, and the defect was filled with an autologous cancellous bone graft from the iliac crest. The postoperative recovery was uneventful, and classic X-ray showed proper plate position (Figures 4 and 5).

**Figure 6:**
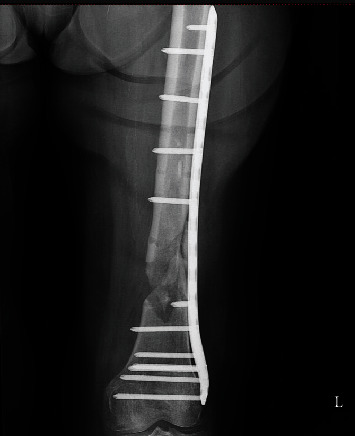
The nonunion is widened, with the plate providing stability in AP view.

**Figure 7:**
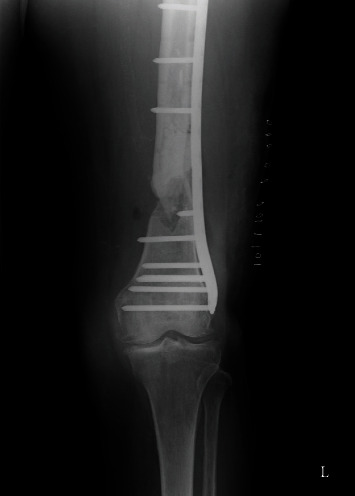
The first stage of the induced membrane technique (IMT), the nonunion is partially filled with PMMA cement. The cement is encircled.

Six months after the procedure, on a follow-up visit, the patient complained about pain around the knee. X-rays showed a halt in bone healing (Figure 6), and another procedure was recommended. The first stage of the induced membrane technique (operation of Masquelet) with a poly-methyl methacrylate (PMMA) cement spacer was performed: the fracture fragments were debrided, and the now widened fracture site was filled with cement (PMMA) (Figure 7).

The second stage was scheduled in two months. During the second stage, PMMA bone cement was extracted from the fracture site, and one screw was removed from the plate to lengthen the segment of the plate which is able to bend and therefore prevent breakage. Additionally, an autologous cancellous graft from the proximal tibia mixed with calcium phosphate (Ca-P) cement (Innotere GMBH) was placed in the interfragmentary space.

**Figure 8:**
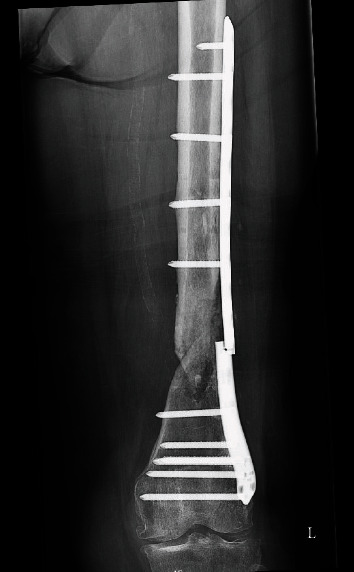
The nonunion is readily visible, and the LCP has failed in AP view.

Tissue samples from the nonunion site were taken. The postoperative recovery was complicated by an episode of fever and elevation of laboratory inflammation markers which was treated successfully with antibiotics. The microbial analysis of the intraoperative fracture site samples confirmed the presence of coagulase-negative *Staphylococcus*, which was treated with antibiotics: ciprofloxacin and clindamycin. Ten months after the last procedure, the was patient presented in our emergency room. She complained about the sudden onset of pain in the left femur during walking, and X-ray confirmed plate breakage (Figure 8).

**Figure 9:**
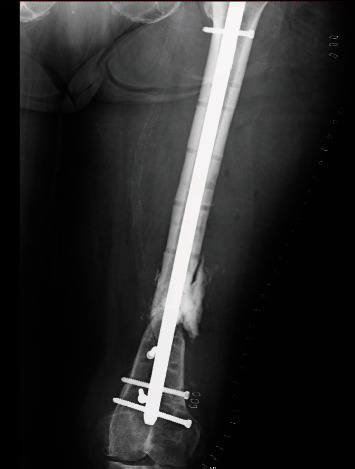
The plate was removed, the nonunion was stabilized with a locked intramedullary nail (the position of the nail is secured with Poller blocking screws (marked by arrows)), and the defect was filled with a mixture of cancellous bone graft and Ca-P cement (encircled).

The patient was admitted to the hospital again and prepared for surgery: during surgery, the broken plate was extracted, and a locked femoral nail (Stryker) was inserted guided by blocking (Poller) screws. The defect was filled with calcium phosphate cement (Innotere GMBH) (Figure 9).

Again, microbial samples were taken, and *Corynebacterium macginleyi* was detected which was treated with targeted antibiotic therapy. The surgical wound showed signs of infection in the form of minimal serous secretion. The wound was dressed weekly. In one of the regular follow-ups, the patient presented with a tumefaction in the lateral thigh at the site of the surgical wound. A puncture was performed, serous fluid was evacuated, and a sample was sent for microbial analysis. Classic X-ray detected the cause of the tumefaction: a part of the cement dislocated from the fracture site into the surrounding tissue.

**Figure 10:**
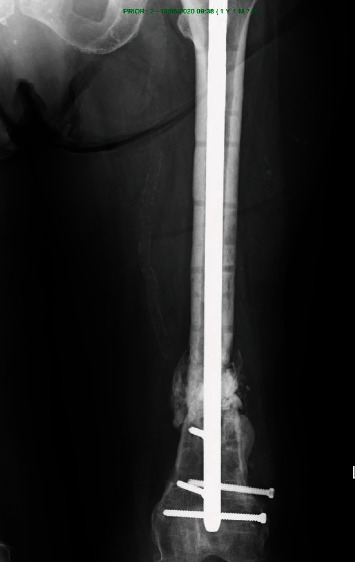
Healing of the nonunion after nailing and dynamization in a favorable position is evident, AP view.

**Figure 11:**
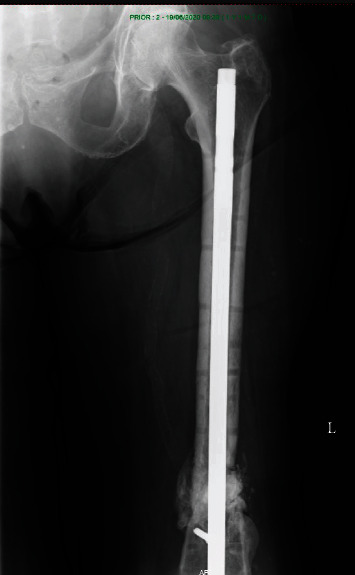
Healing of the nonunion after nailing and dynamization in a favorable position is evident, and the tip of the nail does not protrude into the abductor muscles, AP view.

**Figure 12:**
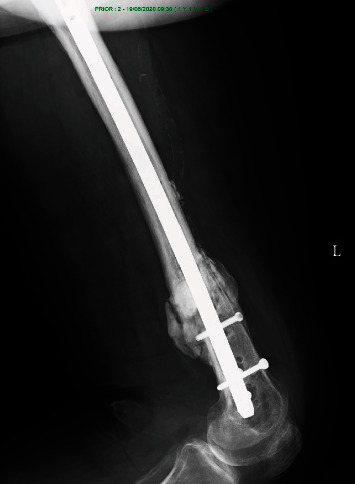
Healing of the nonunion after nailing and dynamization in a favorable position is evident, LL view.

Again, the patient was hospitalized for surgery. Secondary dynamization of the femoral nail was performed, and the dislocated cement was removed from the soft tissue. Follow-up was regular, and bone healing was uneventful (Figures 10–12).

One year after the last procedure and three and a half years after the first procedure for the distal femoral fracture, the patient has much less pain and can walk without assistance. Leg length discrepancy was 1.5 cm shorter on the left side.

## 3. Discussion

The allure of distal femur fracture surgery is the multiple options the surgeon can deploy for a particular case, while knowing that out of the many options, only one is usually the most appropriate.

This paper demonstrates our approach to this very complex case and how we managed all the problems we encountered along the way with the resources and implants we had at our disposal.

Historically, from the 1960s, onward distal femur fractures were treated conservatively with fracture bracing and traction with success rates up to 67-90% [8]. Only with the development new surgical techniques and technologies the approach has shifted from conservative to surgical stabilization. Especially advances in the understanding of the anatomy and biology of distal femur fractures have led to the introduction of new techniques optimizing surgical treatment.

The fundamental goal of surgical stabilization is to achieve the best as possible reduction and maximal stability. If the risk of nonunion is clearly present, it is recommended to use autologous bone grafts to improve stability and optimize the healing process [9].

Today, the most popular surgical options for distal femur fractures are locking plating (LCP) and retrograde intramedullary nailing (RIMN) with our without a bone graft [3]. These two methods have shown better results than nonlocking plates [10] with minimal differences between each other [11]. Nevertheless, both procedures have nonunion rates from 0% to 34% [1, 4].

In this case report, the following risk factors for nonunion were present: female sex, old age, and diabetes, as well as inappropriate surgical techniques. The authors think that the initial treatment should be criticized for implant selection. Complex cases such as this with an established risk for nonunion call for a fixation system that renders absolute stability while avoiding excessive rigidity of the system itself, for which the “classic” plate fixation is known for. Additionally, we believe that the shortness of the plate and screw positioning compromised the stability of the implant which led to the nonunion and fatigue failure of the implant.

In retrospect, the authors believe that the LCP was also not an appropriate option for the same reason as the “classic” plate fixation. Gautier and Sommer recommend LCP only as bridging plates in achieving relative stability [12] which when analyzing our case makes perfect sense. LCPs have become the predominant treatment option of distal femur fractures because of the high stability afforded, and the minimal amount of periosteal stripping makes the technique less disruptive and invasive but also less technically demanding [13]. Another advantage is their ability to gain fixation in the very distal articular fragments [14]. Despite the advantages, distal femoral fractures treated with LCPs are associated with significant complications: from implant failure to delayed and eventually nonunion [15]. Different reviews with up to 31 cases of distal femur fracture nonunion have shown that LCPs are unfortunately rarely the definitive treatment option [16–18].

After the failure of the LCP technique, one option was the ring external fixator first developed by Ilizarov to achieve bone transport [19]; however, we do not have access to the Ilizarov external fixator. Another possible approach was to use a fibular strut either autogenous or from a bone bank which provides a degree of immediate stability while being osteoinductive and osteoconductive.

Eventually, the decision in our case fell for the Masquelet technique, which in our case has been found to be effective in achieving union. The Masquelet technique, first reported by Masquelet in 2000 [20], is also called the induced membrane technique (IMT). It has been used to reconstruct larger diaphyseal defects. It is a two-stage procedure. During the first stage procedure, an extensive debridement of the site and stabilization of the fracture is performed. The next step is the insertion of a poly-methyl methacrylate (PMMA) spacer into the bone defect. The spacer is thought to act as a foreign body, and its goal is to induce the formation and maturation of a biological membrane encapsulating the bone defect. In the second stage, preferably after 4-8 weeks, the spacer is removed through a longitudinal incision to preserve the biological membrane; and in its place, an autologous cancellous bone graft is placed. The key of the procedure is the induced membrane akin to the periosteum. It is highly vascularized and contains osteogenic cells and growth factors [21]. It forms a closed environment stimulating bone regeneration while at the same time limiting the resorption of autologous bone graft when present. Additionally, it acts as a physical barrier preventing the intrusion of soft tissue and providing a degree of mechanical stability. Today, no clear consensus exists on the optimal approach for this technique, while many different modifications and advancements have been reported [22]. Likewise, outcomes differ largely between those variations [23]. But it is, in general, an effective technique to achieve infection eradication and bone union in around 80% of cases [24].

In this particular case, the authors believe that the combination of the induced membrane technique (Masquelet procedure) as a biological stimulus and the stability provided by the locked intramedullary femoral nail was crucial in obtaining bone healing. The authors believe that we have accomplished the goal to restore the axis of the lower limb when weight bearing without disruption of the extensor apparatus [25].

We would like to draw attention to the Ca-P cement dislocation that developed postoperatively. This rare complication was not reported earlier in the literature after the Masquelet procedure which makes our case even more unique. It is important to perform an X-ray for tumefactions at the operation wound to rule out this very rare complication.

This brief summary explores the alternatives that could have been used and illustrates the aforementioned multiple options the surgeon has when dealing with this challenge.

Another very important aspect of patient management in these cases is the antibiotic regimen, and the duration of treatment which is beyond the orthopedic and trauma surgeon and consultation with an infectious disease specialist is paramount. The same is true for dealing with complications of antibiotics.

## 4. Conclusion

This paper illustrates the significance of adequate primary surgery because revisions are always much more complicated; however, even in these challenging circumstances, the basic principles are still true. A solid grasp of these principles allows the surgeon to tailor the treatment plan according to the patient and technical abilities to achieve a satisfactory end result and overcome any complications that may arise in this long and difficult journey for the patient and the surgeon.

## Data Availability

Not applicable.

## Abbreviations

RIMN: Retrograde intramedullary nailing
LCP: Locking compression plate
IMT: Induced membrane technique
PMMA: Poly-methyl methacrylate cement.

## References

[1] Gwathmey F. W. J., Jones-Quaidoo S. M., Kahler D., Hurwitz S., Cui Q. (2010). Distal femoral fractures: current concepts. *The Journal of the American Academy of Orthopaedic Surgeons*.

[2] Martinet O., Cordey J., Harder Y., Maier A., Bühler M., Barraud G. E. (2000). La epidemiologia de las fracturas del femur distal. *Injury*.

[3] Pogliacomi F., Artoni C., Calderazzi F. (2020). Distal femur nonunion treated with retrograde intramedullary nailing and RIA: a case report. *Acta Bio-Medica*.

[4] Ricci W. M., Streubel P. N., Morshed S., Collinge C. A., Nork S. E., Gardner M. J. (2014). Risk factors for failure of locked plate fixation of distal femur fractures: an analysis of 335 cases. *Journal of Orthopaedic Trauma*.

[5] Salduz A., Kaya Ö., Balci H. İ. (2016). Surgical treatment of an infected nonunion of the middle third of the femur associated with femoral shortening in a hemophilia patient. *Case Reports in Orthopedics*.

[6] Ebraheim N. A., Buchanan G. S., Liu X. (2016). Treatment of distal femur nonunion following initial fixation with a lateral locking plate. *Orthopaedic Surgery*.

[7] Agha R. A., Borrelli M. R., Farwana R. (2018). The SCARE 2018 statement: Updating consensus Surgical CAse REport (SCARE) guidelines. *International Journal of Surgery*.

[8] Sain A., Sharma V., Farooque K., V M., Pattabiraman K. (2019). Dual plating of the distal femur: indications and surgical techniques. *Cureus*.

[9] Gangavalli A. K., Nwachuku C. O. (2016). Management of Distal Femur Fractures in Adults: An Overview of Options. *Orthopedic Clinics of North America*.

[10] Meneghini R. M., Keyes B. J., Reddy K. K., Maar D. C. (2014). Modern retrograde intramedullary nails versus periarticular locked plates for supracondylar femur fractures after total knee arthroplasty. *The Journal of Arthroplasty*.

[11] Magill H., Ponugoti N., Selim A., Platt J. (2021). Locked compression plating versus retrograde intramedullary nailing in the treatment of periprosthetic supracondylar knee fractures: a systematic review and meta-analysis. *Journal of Orthopaedic Surgery and Research*.

[12] Gautier E., Sommer C. (2003). Guidelines for the clinical application of the LCP. *Injury*.

[13] Kubiak E. N., Fulkerson E., Strauss E., Egol K. A. (2006). The evolution of locked plates. *The Journal of Bone and Joint Surgery*.

[14] Rodriguez E. K., Boulton C., Weaver M. J. (2014). Predictive factors of distal femoral fracture nonunion after lateral locked plating: a retrospective multicenter case-control study of 283 fractures. *Injury*.

[15] Henderson C. E., Kuhl L. L., Fitzpatrick D. C., Marsh J. L. (2011). Locking plates for distal femur fractures: is there a problem with fracture healing?. *Journal of Orthopaedic Trauma*.

[16] Gardner M. J., Toro-Arbelaez J. B., Harrison M., Hierholzer C., Lorich D. G., Helfet D. L. (2008). Open reduction and internal fixation of distal femoral nonunions: long-term functional outcomes following a treatment protocol. *Journal of Trauma and Acute Care Surgery*.

[17] Bellabarba C., Ricci W. M., Bolhofner B. R. (2001). Results of indirect reduction and plating of femoral shaft nonunions after intramedullary nailing. *Journal of Orthopaedic Trauma*.

[18] Chapman M. W., Finkemeier C. G. (1999). Treatment of supracondylar nonunions of the femur with plate fixation and bone graft. *The Journal of Bone & Joint Surgery*.

[19] Bhardwaj R., Singh J., Kapila R., Boparai R. S. (2019). Comparision of Ilizarov ring fixator and rail fixator in infected nonunion of long bones: a retrospective followup study. *Indian journal of orthopaedics*.

[20] Masquelet A. C., Fitoussi F., Begue T., Muller G. P. (2000). Reconstruction of the long bones by the induced membrane and spongy autograft. *Annales de chirurgie plastique et esthetique*.

[21] Pelissier P., Masquelet A. C., Bareille R., Pelissier S. M., Amedee J. (2004). Induced membranes secrete growth factors including vascular and osteoinductive factors and could stimulate bone regeneration. *Journal of orthopaedic research*.

[22] Giannoudis P. V., Faour O., Goff T., Kanakaris N., Dimitriou R. (2011). Masquelet technique for the treatment of bone defects: Tips-tricks and future directions. *Injury*.

[23] Morelli I., Drago L., George D. A., Gallazzi E., Scarponi S., Romanò C. L. (2016). Masquelet technique: myth or reality? A systematic review and meta-analysis. *Injury*.

[24] Fung B., Hoit G., Schemitsch E., Godbout C., Nauth A. (2020). The induced membrane technique for the management of long bone defects. *The Bone & Joint Journal*.

[25] Ryan J. A., Meyers K. N., DiBenedetto P., Wright T. M., Haas S. B. (2010). Failure of the patellar tendon with the patella everted versus noneverted in a matched-pair cadaver model. *HSS Journal*.

